# The promise of pulsed field ablation and the challenges ahead

**DOI:** 10.3389/fcvm.2023.1235317

**Published:** 2023-10-23

**Authors:** Shruti Krishna Iyengar, Sumedh Iyengar, Komandoor Srivathsan

**Affiliations:** The Division of Cardiovascular Diseases, Mayo Clinic Hospital, Phoenix, AZ, United States

**Keywords:** atrial fibrillation, radiofrequency ablation, pacemaker, mitral valve repair, pulse field ablation

## Abstract

For many years, guidelines have suggested thermal ablation for the treatment of atrial fibrillation. Thermal ablation involves the destruction of tissue, leading to multiple complications. This ablation technique has been tried and tested, however, newer techniques are being investigated in order to avoid these complications. Pulsed field ablation, a nonthermal method of tissue ablation, is being explored as a more safe and efficient way to treat atrial fibrillation. This mini review aims to highlight the mechanisms of pulsed field ablation, its history and evolution, previous studies showing its efficacy, its major challenges and pitfalls, and future advancements to overcome these challenges. This method of ablation could potentially revolutionize the treatment of atrial fibrillation and prevent recurrences, thereby making it easier for the physicians and patients involved.

## Introduction

Pulsed-field ablation (PFA) is a nonthermal method of tissue ablation technology that utilizes high-amplitude pulsed electrical fields to create irreversible electroporation (IRE) in tissues. Unlike traditional thermal ablation technologies, PFA does not rely on heating to damage and destroy tissue. Instead, PFA creates nanopores in cell membranes due to transient, high-voltage exposure that disrupts cell wall integrity, which leads to cell death (1).

Adverse events such as atrioesophageal fistula formation, phrenic nerve injury, stroke, cardiac tamponade, and pulmonary vein stenosis may complicate traditional thermal ablation (2). The risk of thermal ablation procedures is small, but when they occur risk of injury to the esophagus, phrenic nerve, and pulmonary veins ranges from 0.5% to 5%. In contrast, pulsed-field ablation creates non-thermally cardiac lesions in seconds, causing irreversible electroporation (3). In this mini review, we hope to bring to light successful trials involving PFA, the many advantages of PFA over traditional ablation methods, to discuss its challenges as well as its many potentials in cardiovascular medicine.

## History and evolution of pulsed field ablation

In 1979, during an electrophysiological study, direct external current (D.C.) cardioversion triggered a complete atrioventricular (A.V.) conduction block. In preclinical animal studies, high-energy D.C. shock delivered through a conventional diagnostic catheter positioned at the His bundle region was shown to produce a complete A.V. block. The “closed-chest catheter ablation” procedure was then developed to treat drug-resistant supraventricular tachycardias. It was explored for ablation of other conditions like accessory A.V. pathways, atrial tachycardias, and life-threatening VT (4).

D.C. ablation was abandoned in the 1990s due to serious complications like cardiac perforation, heart failure, lethal ventricular arrhythmias, and sudden cardiac death. The international registry on D.C. ablation of V.T. observed a high mortality rate of up to 25%. These complications were attributed to barotrauma caused by using high-energy D.C. and a small ablation electrode in conventional diagnostic catheters (4).

Due to these reasons, for years now, small electrode, radiofrequency catheter-mediated thermal ablation has become common. Alternatively, a catheter or balloon-based cryo ablation is also used to freeze and crystallize intracellular water to cause tissue disruption. The recommended long-term treatment for paroxysmal symptomatic atrial fibrillation has been pulmonary vein isolation (PVI), which involves using thermal catheter ablation to isolate arrhythmogenic foci within the pulmonary veins. However, these ablation techniques can cause collateral damage to cardiac and extracardiac tissue, leading to major complications (5).

D.C. ablation is now being reconsidered for cardiac ablation due to catheter and delivery technology advancements. The latest catheter designs can deliver lower energy and current density due to waveform and frequency, preventing arc formation. A newly developed capacitive power source provides less energy in a shorter period, avoiding high peak current and voltage, thereby eliminating the risk of barotrauma. Clinical studies have shown that lower energy levels can effectively create myocardial lesions for ablation (4).

This method is now known as “pulsed field ablation” or PFA. It uses ultra-rapid electric fields to create nanoscale pores in the target tissues, leading to cell death. PFA potentially creates full transmural lesions in the atrial myocardium while avoiding damage to adjacent tissues and structures, making it a promising alternative to traditional thermal ablation methods (5). This is due to clinically significant variability in susceptibility to high voltage gradient fields.

This method uses irreversible electroporation and is a nonthermal ablation modality investigated since 2011 for creating myocardial lesions. Preclinical studies have shown that PFA can create deep myocardial lesions without causing clinically relevant damage to extracardiac tissue. Clinical studies since 2018 have reported effective and safe ablation using PFA for PVI in patients with paroxysmal atrial fibrillation. In January 2021, the first endocardial ablation system received commercial approval for cardiac tissue ablation to treat paroxysmal atrial fibrillation (4).

## Mechanism of pulsed field ablation

**Figure 1:**
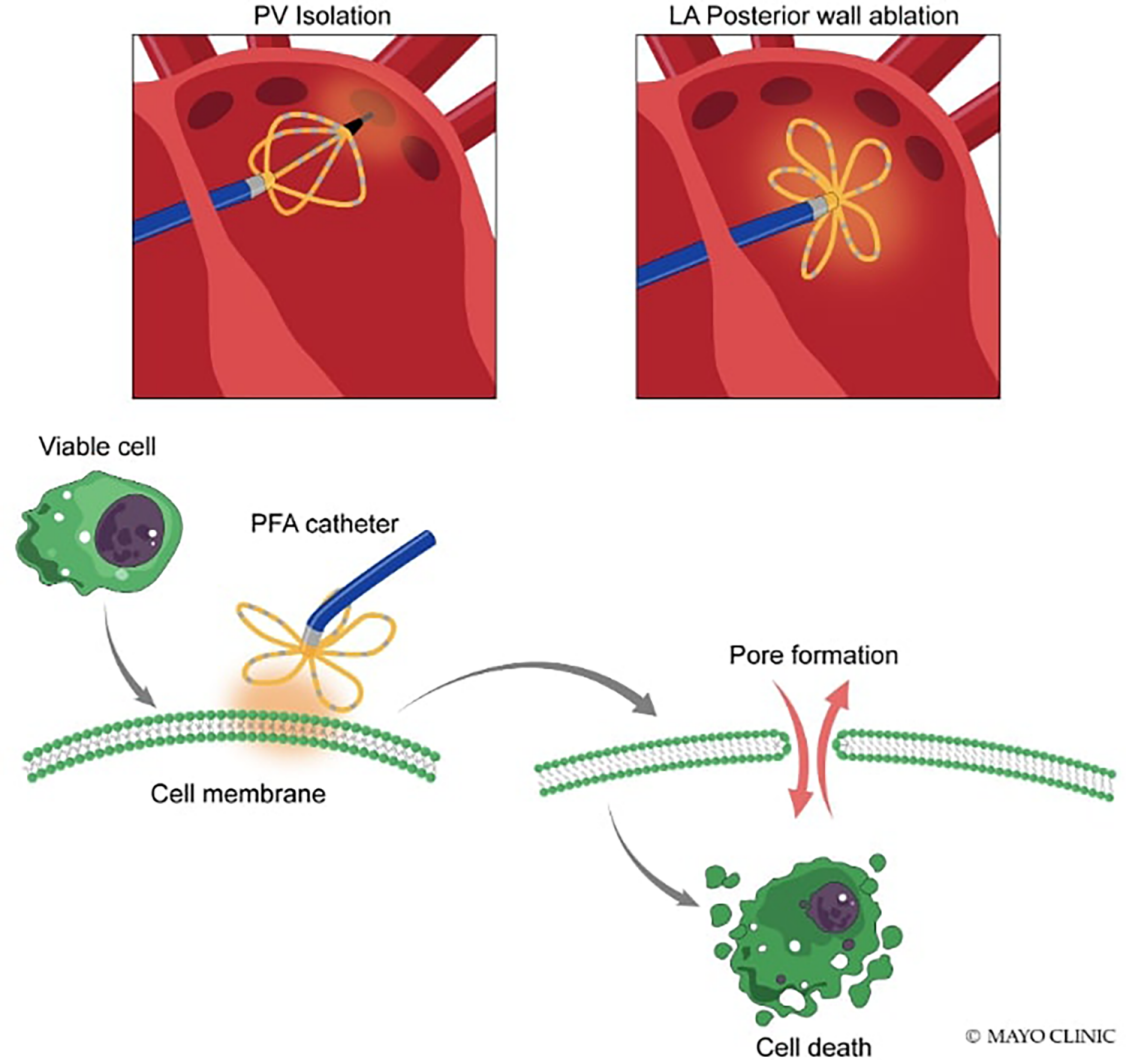
Mechanism of electroporation.

PFA is a nonthermal technique to ablate cells. In PFA, high electric field gradients are applied to cardiomyocytes using D.C. energy, which is rapidly pulsed. This process creates small pores in the cell membrane, known as electroporation, which makes the membrane more permeable. The strength of the electric field applied determines whether the effect is reversible or irreversible, with reversible electroporation repairing cell membranes and greater electric field application causing irreversible cell death through apoptosis or necrosis (Figure 1) (6).

This irreversible electroporation does not cause significant protein denaturation or damage to tissue scaffolding (3). It appears to be particularly well-suited for cardiac ablation, as cardiac cells have one of the lowest threshold values of any tissue (7). PFA uses ultrarapid electrical pulses to ablate myocardial tissue while sparing surrounding tissue preferentially. It has a lower threshold for dielectric cell membrane breakdown resulting in necrosis for the myocardium than surrounding tissues, making it suitable for cardiac ablation. PFA has been shown to spare the esophagus, blood vessels, pericardium, and nerves, resulting in few major complications in preclinical and clinical studies. PFA has received considerable interest for A.F. catheter ablation to improve safety by decreasing collateral damage and improving lesion durability (8).

While some cell types, such as those found in nerves and the esophagus, show greater resistance to change, preclinical studies have proposed that PFA can have a preferential effect on cardiomyocytes over pulmonary venous tissue and myelinated nerve cells. In clinical trials, PFA is an effective and time-efficient method for achieving pulmonary vein isolation without causing collateral damage to surrounding tissues. Furthermore, deliberate ablation of collateral tissues with PFA in preclinical studies did not result in significant injury, and initial pilot human data has been promising. Unlike other energy sources, PFA allows for the adjustment of multiple parameters, leading to various lesion profiles and levels of efficacy (3, 7).

## Animal studies

Wittkampf et al. conducted a study on ten swine to investigate the impact of PFA on the creation of pulmonary vein (P.V.) lesions for PVI. They utilized ablation catheters shaped thermally to resemble the standard 20-mm circular mapping catheter and cooled down in a customized mold. The researchers performed ablation using a non-arcing 200 J application, with a maximum of 4 applications used per P.V. ostia to create P.V. ostial lesions. Following a 3-week follow-up period, they reported a reduction in electrograms at P.V. Ostia, an increase in stimulation threshold, and no signs of P.V. stenosis. Upon histological analysis, they found that the lesions at the sites of P.V. ostia were up to 3.5 mm in depth. Furthermore, they demonstrated the ability to spare the phrenic nerve despite ablation in the immediate vicinity. However, some drawbacks were observed, including difficulty titrating IRE as easily as R.F. and the need for anesthesia. These drawbacks may have to be weighed against the expected shorter procedure time (9).

In an *in vivo* study by Koruth et al., the effectiveness of bipolar PFA was investigated using optimized monophasic and biphasic waveforms in two groups of seven swine each. The control group used R.F. as the ablation method, and 46 veins were targeted. After ten weeks, the study assessed the electrical isolation of veins acutely and through histological analysis. Among the 28 veins evaluated for durability, the biphasic form of PFA was significantly safer and more durable (18/18 vs. 10/18 in the monophasic form, *p* = 0.02), indicating that it is a superior method. The study also found that R.F. was associated with nerve damage and P.V. stenosis, while these issues were not observed with PFA (10).

## Initial pilot studies on safety/efficacy

The PULSED AF Pilot Trial utilized a new PFA system for the first time in a human pilot study to determine its acute procedural outcomes. The study found that the PFA system successfully achieved intraprocedural PVI in all 38 patients without any severe adverse events attributed to the system during the 30-day follow-up period. The study suggests that the PFA system's mechanism for lesion creation is mostly nonthermal, as there was little change in luminal temperature during direct PFA delivery over the esophagus. Moreover, the study did not report any complications related to phrenic nerve injury or symptoms of esophageal injury. Compared to traditional thermal catheter ablation, the PFA system requires a significantly shorter duration of energy application to achieve tissue damage, resulting in a shorter procedure time. Although this was only a pilot study with few patients, the PFA system showed promising results. A larger clinical trial called the PULSED AF clinical trial was conducted later to confirm these findings with longer-duration outcomes (Table 1) (11).

**Table 1:** Clinical studies on pulsed field ablation.

Clinical study	Type of subjects	Number studied	Acute outcome	Long-term outcome	Complications	References
Verma et al. (2022) (PULSED AF Pilot Trial)	Human	38	Successful PVI achieved in all patients	Only acute outcome assessed in this study.	None	(11)
Verma et al. (2023) (PULSED AF Clinical Trial)	Human	300	Successful PVI achieved in all patients	66.2% one-year success rate achieved	1 patient developed pericardial effusion	(3)
Stewart et al. (2021)	Pig	6	Complete electrical isolation achieved in all subjects	Complete replacement fibrosis achieved 4 weeks after ablation	None	(16)
Wittkampf et al. (2011)	Pig	10	PV ostial lesions created	Following 3-week period—reduction in electrogram in PV ostia, increase in stimulation threshold.	None—sparing of phrenic nerve was demonstrated.	(9)
Koruth et al. (2019)	Pig	14	PVI achieved in all 14 subjects	Biphasic form of PFA was safer and more durable (18/18 vs. 10/18 in the monophasic form.)	None—no evidence of PV stenosis or nerve damage as opposed to RFA.	(10)
Reddy et al. (2019)	Human	81	All PVIs achieved by either monophasic or biphasic PFA.	Durability of PV isolation improved from 18% to 100% at the 3-month mark.	One case of pericardial tamponade reported	(8)
Yavin et al. (2020)	Pig	12	Block achieved in all subjects. Selectivity on cardiomyocytes demonstrated.	Long term outcome not assessed.	Mild edema on esophagus observed in 1 case	(12)
Gunawardane et al. (2021)	Human	20	PVI achieved in all cases. One case of coronary vessel spasm which improved after administration of nitroglycerine.	PFA did not cause any lasting damage to patient's coronary artery; LV ejection fraction remained normal during follow-up.	One case of coronary vessel spasm	(14)
Reddy et al. (2022)	Human	50	Intracoronary nitroglycerine proved to be effective.	Long term outcome not assessed.	Temporary ST-segment depression	(17)
Maury et al. (2023)	Human	2	Block achieved. Thoracic CT showed pulmonary hemorrhage after the ablation.	One month follow up CT showed regression of the initial images.	Pulmonary hemorrhage	(18)
Ekanem et al. (MANIFEST-PF) (2022)	Human	1,758	Success rate of 99.9% in effectively isolating pulmonary veins (PVI).	No long-term complications associated with the esophagus or phrenic nerve observed beyond the patient's hospital stay.	Pericardial tamponade (0.97%), stroke (0.4%), stroke leading to death (0.06%), transient phrenic nerve paresis (0.46%), coronary artery spasm, hemoptysis, persistent extended dry cough	(19)
Musikantow et al. (2023) (Late-breaking Clinical Trial)	Human	121	PVI achieved in study subjects.	Of 110 patients, at post-PFA follow-up of 48 ± 9 months, 76% remained free from AF/AFL/AT.	(Procedural) One pericardial tamponade, one transient ischemic attack, one vascular hematoma.	(13)
Verma et al. (2023) (Late-breaking Clinical Trial)	Human	277	69.4% of paroxysmal and 62.2% of persistent patients showed no atrial arrhythmia (AA) burden; 87.1% of paroxysmal and 81.8% of persistent patients showed AA burden <10%. AA burden averaged a >19-point (clinically meaningful) QoL improvement.		None	(15)

## Other studies

### Studies involving patients with paroxysmal AF

Two trials evaluated the safety and effectiveness of using catheter based PFA for paroxysmal A.F. in 81 patients. All patients had their pulmonary veins isolated using monophasic or biphasic PFA. The durability of P.V. isolation improved from 18% to 100% at the three-month mark as the waveform was refined. Only one case of pericardial tamponade was reported as a procedure-related adverse event. No other major adverse events were observed during the median 120-day follow-up, and the 12-month Kaplan Meier estimate indicated freedom from arrhythmia of 87.4 ± 5.6% (8).

Other studies provided additional evidence to support the results of these studies. Yavin et al. conducted a similar analysis to explore using PFA (biphasic form) with a lattice-tip catheter. They examined the feasibility of creating an atrial line of block and the safety of PFA on the phrenic nerve and the esophagus. They also evaluated the durability and safety of PFA at a 2-week interval. The authors compared the effects of PFA and R.F. ablation on the esophagus using a mechanical deviation model and direct ablation within its lumen. According to their findings, PFA produced an electrical block in 100% of the lines created, confirmed by histological analysis indicating complete transmurality of lesions. PFA had a selective impact on cardiomyocytes while sparing blood vessels and surrounding nervous structures. Mild edema was the only side effect observed on the esophagus with PFA, whereas R.F. ablation caused epithelial ulcerations (12).

One hundred twenty-one patients were enrolled in another late-breaking clinical trial by Musikantow et al., in which the long-term efficacy and safety of PFA after PVI in individuals with PAF were examined. Among the study participants, successful PVI was accomplished, and 110 patients were included in the analysis of long-term outcomes. The results revealed that after a follow-up period of 48 ± 9 months following PFA, approximately 76% of the patients maintained their freedom from AF/AFL/AT (13).

### Studies involving patients with persistent AF

The study conducted by Gunawardene et al. in May 2021 involved 20 patients with A.F. who underwent pulmonary vein isolation guided by PFA, with additional ablation lesions administered at the physician's discretion for persistent A.F. One patient, a 77-year-old man with a history of persistent A.F. and failed antiarrhythmic drug therapy, experienced significant ST-segment elevations on his EKG recordings shortly after PFA applications were used to block the mitral isthmus. An immediate coronary angiogram revealed that the left circumflex artery was occluded due to vessel spasm at the exact location of the prior ablation. However, the occlusion was reversed by administering intracoronary nitroglycerin, and the patient remained stable throughout the procedure, waking up without any chest discomfort or angina pectoris symptoms. Two days later, the right coronary artery was stented, and the spasm of the left circumflex artery was found to have completely resolved. The study concluded that the PFA procedure did not cause any lasting damage to the patient's coronary artery, and his left ventricular ejection fraction remained normal during follow-up (14).

### Studies involving patients with both paroxysmal and persistent AF

The PULSED AF study was conducted between January 2018 and March 2019 to investigate the effectiveness of PFA in treating atrial fibrillation (A.F.) in 300 patients with paroxysmal and persistent symptomatic A.F. that was not responsive to traditional antiarrhythmic drugs. It was the first-in-human clinical trial of its kind. The study monitored patients for a year using ECGs and 24-hour Holter monitoring to assess acute procedural failure, arrhythmia recurrence, and antiarrhythmic escalation. The study found that PFA was effective in 66.2% of patients with paroxysmal A.F. and 55.1% of patients with persistent A.F. after one year. Acute isolation of all P.V.s was achieved in 100% of patients, with an average PFA time of 3 min per patient. One patient with persistent A.F. had a pericardial effusion that needed drainage after undergoing PVI. Overall, the study had a very low incidence of major complications (0.7%) and no cases of esophageal injury, phrenic nerve injury, pulmonary vein injury, or thrombo-embolism-stroke. The P.V. remapping procedures took place after a median of 84 days, and the follow-up period lasted for 120 days (3).

As part of a sub-study of the PULSED AF Trial by Verma et al, a late-breaking clinical trial studied the recurrence of atrial arrhythmia (A.A.) following PFA in patients with A.F. Holter recordings and TTM transmissions were used. 69.4% of paroxysmal and 62.2% of persistent patients showed no atrial arrhythmia (A.A.) burden; 87.1% of paroxysmal and 81.8% of persistent patients showed A.A. burden <10% (15).

During their study, Stewart et al. utilized a circular catheter with nine gold electrodes to deliver low- or high-dose PFA treatments to six pigs in the SVC, RAA, and RSPV. They assessed electrical isolation immediately after the procedure and evaluated chronic lesions through necropsy and histopathology after four weeks. The lesions caused complete electrical isolation in all anatomies and were characterized by being completely circumferential, contiguous, and transmural. Regardless of the endocardial surface structure, they all transformed into consistent lines of chronic replacement fibrosis. The electrodes caused minimal temperature increases after delivery, and no extracardiac damage, stenosis, aneurysms, endocardial disruption, or thrombus was observed. The PFA treatments induced complete circumferential replacement fibrosis four weeks after ablation, and there was an excellent chronic myocardial and collateral tissue safety profile (16).

Reddy et al. conducted a study that found that patients who received PFA to the CTI experienced similar coronary spasm cases, varying in severity across different arterial segments. However, no severe EKG changes were observed, and temporary ST-segment depression was the only noticeable effect. Intracoronary nitroglycerine was effective in relieving the spasm, and administering it before treatment was found to be helpful. However, further research is required to optimize the formulation, route of administration, dose, and timing of nitroglycerin (17).

Another report describes two cases in which patients experienced a possibly serious complication during PFA of the pulmonary veins, in which a small amount of arterial blood was observed in the ventilation system. A thoracic C.T. scan revealed a hemorrhage in both patients' left superior pulmonary lobes. Still, a follow-up C.T. scan one month later showed that the initial images had completely regressed. The bleeding was likely due to a stiff guidewire that could have damaged the distal pulmonary vein vasculature and penetrated the pulmonary alveoli rather than being caused by the pulsed-field energy. The need for anticoagulation during ablation procedures may have contributed to detecting this hemorrhage. It was concluded that using J-tip guidewires may be safer, as 40% of patients undergoing bronchoscopy showed small blood clots (18).

The MANIFEST-PF study across 24 centers used a special catheter (pentaspline PFA) to treat 1,758 atrial fibrillation patients. The catheter had a 99.9% success rate in isolating pulmonary veins, a treatment focus. No lasting esophagus or nerve issues were seen after hospitalization. Rare major complications (1.6%) included pericardial tamponade (0.97%) and stroke (0.4%), one being fatal (0.06%). Minor issues (3.9%) were mainly blood vessel related (3.3%), with some temporary nerve problems (0.46%) and transient ischemic attacks (0.11%). Uncommon problems like coronary artery spasm, cough, and haemoptysis were also seen (0.06% each). The catheter achieved vein isolation safely, but catheter-related risks like tamponade and stroke emphasized need for improvements (19).

**Figure 2:**
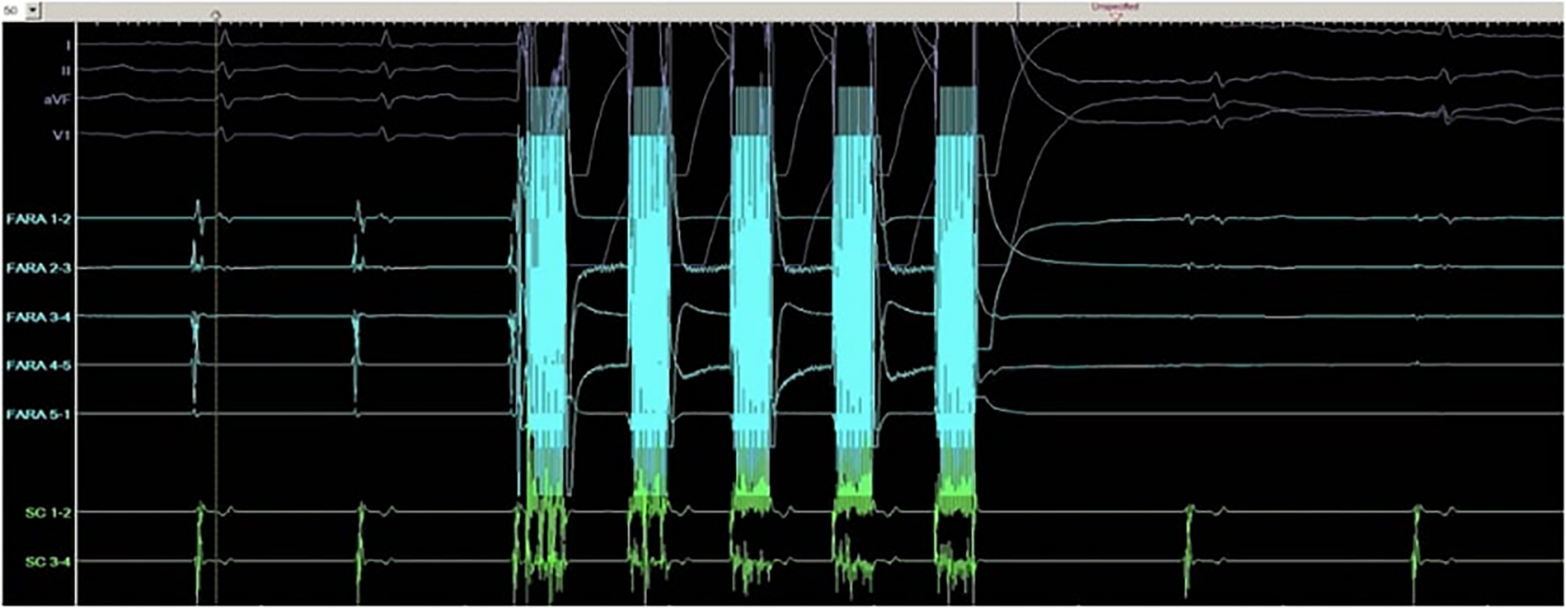
Pulmonary vein isolation tracing with Farapulse pulsed field ablation device.

A recent comprehensive investigation aimed to juxtapose the safety and effectiveness of Pulsed Field Ablation (PFA) with traditional ablative techniques for patients undergoing procedures related to atrial fibrillation (AF). This review encompassed six distinct studies involving a total of 1,897 patients who underwent PFA treatments. The assessment centered on appraising PFA's success in achieving pulmonary vein isolation (PVI), monitoring adverse events, and tracking the recurrence of AF or other irregular cardiac rhythms. The achievement of PVI was widespread, with only a minimal number of exceptions. The analysis underscored PFA's noteworthy success rate, prominently demonstrated in the MANIFEST-PF survey, where the accomplishment of acute PVI reached an impressive 99.9%. Instances of significant complications, such as pericardial tamponade, vascular issues requiring surgical intervention, and strokes, were infrequent. A reduction in the recurrence of atrial arrhythmias was observed in the PFA group (11%) in contrast to the thermal ablation group (39%) (Table 2; Figure 2) (20).

**Table 2:** Pulsed field ablation devices (11, 21–23).

	Medtronic (9)	Boston scientific (21)	Johnson & johnson (22)	Kardium (23)
Year of Study	2021	2019	2020	2019
Device	PulseSelect	Farawave (Farapulse)	Varipulse	Globe
Energy Type	Biphasic, Bipolar waveform	Monophasic (15) and Biphasic (66) PFA	Biphasic, 1,800 Volts	Bipolar and biphasic pulse train
Size of Catheter	Over-the-wire, circular array with 9 gold electrodes. 9F shaft	12F (Over the wire)	7.5F. 10 electrodes. Circular PFA lasso catheter with adjustable diameter between 25 and 35 mm.	Globe Catheter with 122 electrodes. Size of the electrodes ranges from 9.0 to 13.6 mm^2^. Electrodes fanned to form a spherical array with a diameter of 30 mm inside the left atrium.
LA Dwell Time	Average = 82 ± 35 min	23 ± 9 min	82.4 ± 20 min	16 min
Acute Isolation	100%	100%	100%	99.1%
Major Complication(s)	1 patient developed pericardial effusion	Tamponade in 1 patient	No complications.	Pericardial tamponade in 2 patients.
Image	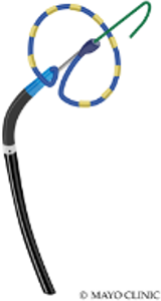	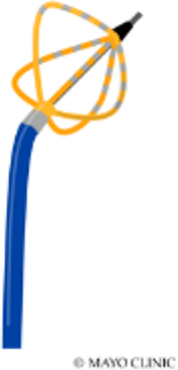	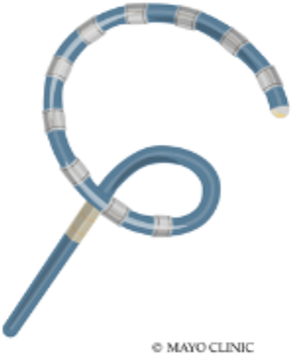	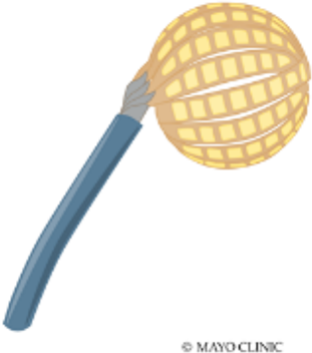

## Challenges of pulsed field ablation

PFA is a promising technique for treating cardiac arrhythmias, but several challenges must be addressed before its widespread adoption in clinical practice. One of the main challenges is the lack of standardized protocols for PFA, including energy delivery parameters, which can vary widely depending on the target tissue and clinical indication, as PFA is a relatively new technique. This variability makes it difficult to compare results across studies and hinders the adoption of PFA (8). Another challenge is the need for improved targeting of the tissue of interest, which can be difficult, especially for structures that are hard to access or have complex anatomies. Recent studies have demonstrated the success of MRI and C.T. image-guided ablation techniques (8). While improved imaging techniques and real-time guidance systems can help overcome this challenge, further development is needed.

Furthermore, PFA has been known to lead to complications such as coronary artery spasms and pulmonary artery hemorrhage (14, 18). Further research is necessary to develop methods to prevent such complications effectively. PFA may also cause collateral damage to surrounding tissues, as Howard B et al. reported a dose-dependent phrenic nerve stunning caused by PFA in his study (24). Lack of standardization of the PFA procedure can further increase these dose-dependent complications. Additionally, the cellular mechanisms underlying the effects of PFA are not yet fully understood, and further research is needed to optimize treatment parameters accordingly.

Another factor to consider is that although there have been studies on the safety and efficacy of PFA devices but there has not been a randomized clinical trial that directly compares the clinical outcomes of PFA with those of existing ablation technologies such as radiofrequency ablation (RFA) or cryoablation (CBA). Furthermore, long-term data on the safety and efficacy of PFA is still limited, and large-scale studies with longer follow-up periods are needed to evaluate the long-term benefits and risks of PFA and to establish its role in clinical practice (25).

Lastly, as PFA is a novel procedure; certain financial factors can limit its widespread use and accessibility to some patients. The equipment cost and the need for specialized training also make it difficult to be adopted by all institutions.

## Future advancements

PFA is a promising technology for treating various medical conditions, including cancer and arrhythmias. By utilizing catheter ablation, PFA can effectively treat paroxysmal and persistent atrial fibrillation (A.F.). However, traditional thermal ablation modes risk damaging nearby tissue and require lengthy treatment times. In contrast, PFA provides a nonthermal approach to inducing cell death, which can lead to faster and safer cardiac ablation, ultimately improving its efficiency and effectiveness (3). PFA shows promise in CTI, posterior wall isolation, and VT ablation. It creates effective transmural lesions without collateral damage in CTI (8, 26). In posterior wall isolation, it's valuable for deeper lesions when standard methods aren't sufficient (27). For ventricular tachycardia, PFA's selectivity for myocardial tissue targets arrhythmogenic foci in ventricular walls (27). As research on PFA continues, several advancements are being made to improve its effectiveness and reduce its limitations. One such development is the miniaturization of PFA catheters, which enables their use in smaller and more complex anatomical structures, allowing for more targeted and precise ablation (28). Optimization of energy delivery parameters is also being investigated, which can result in more efficient and uniform tissue ablation, reducing the need for multiple ablation applications, reducing the damage to pulmonary veins caused by field ablation, and improving procedural efficiency (29). Finally, using artificial intelligence and machine learning algorithms can potentially improve the accuracy and effectiveness of PFA procedures by analyzing imaging data to identify the most suitable targets (30). These advancements are expected to pave the way for the wider adoption of PFA in clinical practice and improve patient outcomes. There must be more studies showing the long-term efficacy of pulsed-field ablation, as there have been no long-term studies.
